# Kinematic-Model-Free Predictive Control for Robotic Manipulator Target Reaching With Obstacle Avoidance

**DOI:** 10.3389/frobt.2022.809114

**Published:** 2022-02-02

**Authors:** Ahmad AlAttar, Digby Chappell, Petar Kormushev

**Affiliations:** ^1^ Robot Intelligence Lab, Dyson School of Design Engineering, Imperial College London, London, United Kingdom; ^2^ Robotics Lab *,* Dubai Future Labs, Dubai, United Arab Emirates

**Keywords:** kinematic-model-free, predictive control, target reaching, obstacle avoidance, adaptive control, model-free control

## Abstract

Model predictive control is a widely used optimal control method for robot path planning and obstacle avoidance. This control method, however, requires a system model to optimize control over a finite time horizon and possible trajectories. Certain types of robots, such as soft robots, continuum robots, and transforming robots, can be challenging to model, especially in unstructured or unknown environments. Kinematic-model-free control can overcome these challenges by learning local linear models online. This paper presents a novel perception-based robot motion controller, the kinematic-model-free predictive controller, that is capable of controlling robot manipulators without any prior knowledge of the robot’s kinematic structure and dynamic parameters and is able to perform end-effector obstacle avoidance. Simulations and physical experiments were conducted to demonstrate the ability and adaptability of the controller to perform simultaneous target reaching and obstacle avoidance.

## 1 Introduction

**FIGURE 1:**
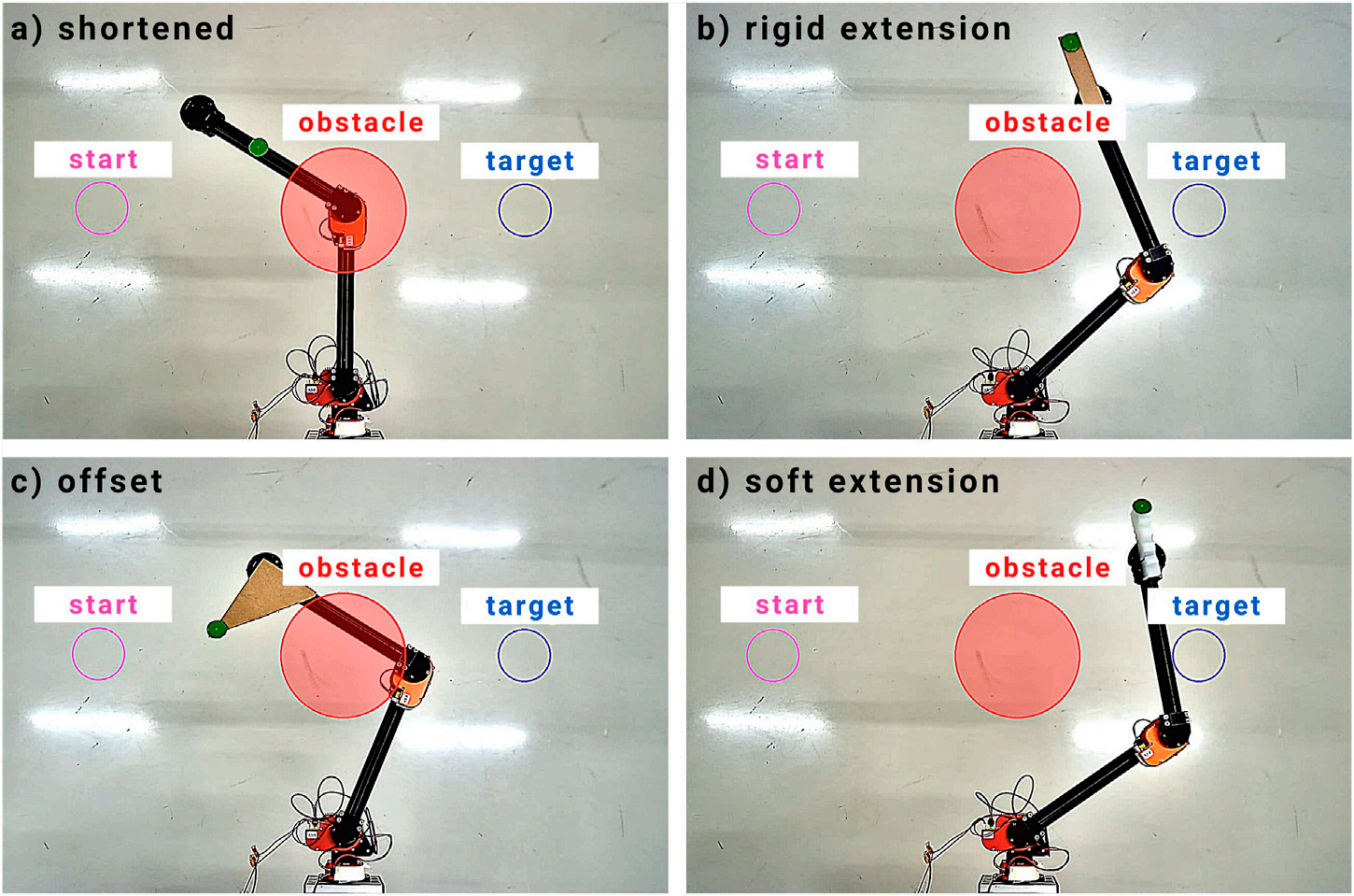
Experimental setup to test the adaptability of the proposed KMF-PC controller. The magenta circle represents the initial point prior to robot motion, the red circle represents a virtual obstacle, and the blue circle represents the target position. The green circle on the robot is the tracked point. Midway reaching, the end effector is modified to demonstrate the adaptability of the controller. Four tests were conducted: **(A)** The end effector was shortened by moving the tracked point down the second link; **(B)** The end effector was lengthen by adding a rigid extension; **(C)** The end effector was offset at an angle; **(D)** The end effector was extended using a soft link.

Researchers over the past few decades studied optimal control, especially for applications where systems dynamics and constraints require proper handling (Zanon et al., 2017). Model Predictive Control (MPC) is an advance control method widely used in academia and industry (Forbes et al., 2015). MPCs are widely used in robotics due to their ability to handle constraints and run in real-time (Saputra et al., 2021). In general, MPCs require a system model prior to control in order to optimize the controller over a finite horizon (Stenman, 1999) which can be challenging to obtain or maintain for certain types of robots (e.g., soft robots, continuum robots, transforming robots, etc.) (AlAttar et al., 2021). The kinematic-model-free (KMF) controller is capable of controlling robot manipulators without any previous knowledge of the robot’s kinematic structure or dynamic properties. The controller works by building local linear models that are used to perform reaching tasks (AlAttar and Kormushev, 2020). However, the KMF controller up to date have not been applied to high-level tasks such as trajectory planning and obstacle avoidance as they are concerned with low-level control. Along with the merits of MPCs, integrated together, they can perform high-level tasks. In this paper, a novel kinematic-model-free predictive controller (KMF-PC), that is capable of performing end-effector reaching and obstacle avoidance for robot manipulators, is presented. The controller was tested in simulation as well as on a physical 2-degrees-of-freedom (2-DOF) manipulator shown in Figure 1.

### 1.1 Contributions

The contributions of the paper are: Presenting a novel model-free controller, KMF-PC, capable of controlling a robot arm without any prior knowledge of the robot’s kinematics or dynamics and achieving end-effector obstacle avoidance using a kinematic-model-free approach. Furthermore, the adaptability of the controller is tested by modifying the end effector during execution.

### 1.2 Paper Structure

The paper is organized as follows: Section 2 introduces work in the literature that is relevant to the research presented, Section 3 presents the formulation of the KMF-PC controller, Section 4 presents simulation and experimental results, and finally Section 5 concludes the paper.

## 2 Related Work

MPC is a control method that uses a system model to predict future behavior which can be sensitive to prediction errors, especially in the case of nonlinear plants. To mitigate such sensitivities, adaptive model predictive control, where the plant model and nominal conditions are continuously adapted, is used (Franco and Santos, 2019). MPCs gained popularity due to its ability to handle constraints and its capacity to run in real-time, which allowed it to be widely used in robotics (Saputra et al., 2021). MPCs have been used for path planning and collision avoidance of road vehicles (Ji et al., 2016) and multiple micro aerial vehicles (Kamel et al., 2017). Krämer et al. (2020) controlled a manipulator to perform reaching tasks while avoiding a dynamic obstacle. Nikou et al. (2017) used a nonlinear-MPC to control *N* robots for cooperative manipulation of objects whilst avoiding obstacles. Li and Xiong (2019) performed obstacle avoidance of mobile manipulators using an MPC.

Li et al. (2021) used MPC for trajectory tracking and obstacle avoidance of mobile robots. Oleinikov et al. (2021) used MPC for controlling a robot manipulator that avoids fixed and human obstacles. Ashe and Krishna (2020) used MPC for dynamic target tracking and obstacle avoidance for a person-following robot. Pankert and Hutter (2020) used MPC for continuous mobile manipulation and collision avoidance. Other researchers used MPC for controlling an autonomous vehicle and performing obstacle avoidance (Quirynen et al., 2020; Song and Huh, 2021). Wang et al. (2021) used a MPC for controlling a dog robot for helping vision-impaired people to navigate around obstacles.

In general, MPCs require a system model for predicting the state of the system over a finite horizon Dao et al. (2021). This imposes that the system to be controlled and its dynamic model must be known prior to control. Furthermore, nonlinear models can burden the controller with heavy computational requirements (Stenman, 1999). This can be challenging in robotics as some robots, such as soft and continuum robots, are not straightforward to model accurately, while others might change, adapt, morph, transform, or evolve, such as robots that change their kinematic structure or dynamic properties during movement or robots that get accidentally damaged or bent (AlAttar et al., 2021).

On the other hand, KMF control methods can overcome these challenges as these control methods do not require any prior knowledge of the robot’s kinematic or dynamic models. Kinematic-model-free controllers work by gathering information through short exploratory actuations, building a local linear model that approximates the robot’s local kinematic and dynamic behaviour, and estimating an actuation signal that moves the robot towards a given reference target. In our previous work, we’ve demonstrated KMF position control of a robot manipulator capable of performing target-reaching tasks (Kormushev et al., 2015a, Kormushev et al., 2015b). The controller was then experimented on a pneumatically-actuated soft continuum robot (Frazelle et al., 2021) for space applications. We, then, presented a pose (position and orientation) controller that controls a robot’s end-effector pose using locally weighed dual quaternions (AlAttar and Kormushev, 2020). Finally, we scaled up the degrees-of-freedom to be controlled by tracking and controlling multiple points along the robot’s kinematic chain (AlAttar et al., 2021).

Although model-free predictive control methods were previously studied (Stenman, 1999; Yamamoto, 2014; Li and Yamamoto, 2016), online kinematic-model-free predictive control for robot manipulators is an unexplored area of research up to our knowledge.

## 3 Problem Formulation

This section formulates the KMF-PC controller. The nonlinear model of a rigid-body robot manipulator can be obtained using the following general dynamic equation:

$$\begin{array}{l} M\left(q\left(t\right)\ddot{q}+c\left(q,\dot{q}\right)+g\left(q\left(t\right)\right)+\tau_{f}\left(t\right)=\tau \left(t\right)\right. \end{array}$$ (1)

where *q* are the joint angles, $\dot{q}$ are the joint velocities, $\ddot{q}$ are the joint accelerations, *M* is the inertia matrix, *c* is the Coriolis vector, *g* is the gravity vector, *τ*_*f*_ is the friction torque, and *τ* is the applied torque.

### 3.1 Kinematic-Model-Free Control

For a 2-DOF robot manipulator, let $\hat{p}$ be the desired actuation primitive whose parameters $\tau_{p}\left(\hat{p}\right)$ are to be estimated to actuate the end effector towards the target position:

$$b_{1}=\left[\begin{array}{c} \tau_{p}^{1}\left(\hat{p}\right)\\ \tau_{p}^{2}\left(\hat{p}\right) \end{array}\right]$$ (2)

Following the kinematic-model-free approach, *b*_1_ can be approximated as a linear combination of actuation primitives as follows:

$$A_{1}w=b_{1}$$ (3)

where $w={\left[w_{1},\;w_{2},\;\ldots ,\;w_{k}\right]}^{T}$ is the vector of unknown weights and *A*_1_, the actuation matrix, is defined as follows:

$$A_{1}=\left[\begin{array}{cccc} \tau_{p}^{1}\left(p_{1}\right) & \tau_{p}^{1}\left(p_{2}\right) & \ldots & \tau_{p}^{1}\left(p_{k}\right)\\ \tau_{p}^{2}\left(p_{1}\right) & \tau_{p}^{2}\left(p_{2}\right) & \ldots & \tau_{p}^{2}\left(p_{k}\right) \end{array}\right]$$ (4)

During exploration, the controller applies pseudo-random actuation primitives with the following curve:

$$\tau \left(t\right)=\left\{\begin{array}{cc} \tau_{p} & \;t\in \left[t_{0},t_{0}+\frac{d_{p}}{2}\right)\\ 0 & \;otherwise \end{array}.\right.$$ (5)

The resulting end-effector displacements are recorded into the observation matrix:

$$A_{2}=\left[\begin{array}{cccc} \Delta x\left(p_{1}\right) & \Delta x\left(p_{2}\right) & \ldots & \Delta x\left(p_{k}\right)\\ \Delta y\left(p_{1}\right) & \Delta y\left(p_{2}\right) & \ldots & \Delta y\left(p_{k}\right)\\ \Delta z\left(p_{1}\right) & \Delta z\left(p_{2}\right) & \ldots & \Delta z\left(p_{k}\right) \end{array}\right]$$ (6)

where Δ*i* (*p*_*j*_) is the displacement effect on the end effector along the *i*-axis as a result of exerting actuation *p*_*j*_. In the planar case, Δ*z* (*p*_*j*_) = 0. Let the desired effect be expressed as follows:

$$b_{2}=\left[\begin{array}{c} \Delta x\left(\hat{p}\right)\\ \Delta y\left(\hat{p}\right)\\ \Delta z\left(\hat{p}\right) \end{array}\right]$$ (7)

The desired effect, *b*_2_, can be expressed as a linear combination of the kNN observed effects, *A*_2_, as follows:

$$A_{2}w=b_{2}$$ (8)

Note that many solutions might exists for *w* since *A*_2_ is might not be full rank. To resolve this issue, least squares regression is used to find the smallest squared vector *b*_2_ that is a solution to the equation. This calculated value can then be used in Eq. 3 to find the desired actuation primitive that will actuate the end effector towards the desired target position. After every actuation, the actual and desired end-effector positions are compared. In the case of significant discrepancy, another exploratory phase is triggered.

### 3.2 Model Predictive Control

The system model of linear MPC in state space form is as follows:

$$\begin{array}{ccc} x\left(k+1\right)=Ax\left(k\right)+Bu\left(k\right) & & \end{array}$$ (9)

$$\begin{array}{ccc} y\left(k\right)=Cx\left(k\right)+Du\left(k\right) & & \end{array}$$ (10)

where matrices *A*, *B*, *C*, and *D* can vary with time and:

•k: current control time index
•x: system state variables
•u: manipulable inputs
•y: system outputs

In general, a system model is used to predict the future states of a dynamic system by the MPC to generates a control signal that minimizes some cost function over a predefined prediction horizon (Afram and Janabi-Sharifi, 2014). The cost function optimizes the control variables to achieve some desirable reference which results in a sequence of *N* optimal actuations, of which the MPC only applies the first (Khosravi et al., 2019). This process is then repeated after shifting the horizon forward. If the system model is linear, analytical solutions to the MPC can be used for extremely fast computation. In the case of rigid-body robots, the dynamic model shown in (Eq. 1) is integrated in time, and becomes extremely non-linear as more joints are added, making MPC challenging. The problem is often simplified by linearizing the system dynamics, however this requires an accurate model of the system; something that is not possible for unknown robots.

### 3.3 Kinematic-Model-Free Predictive Control

To make use of linear MPCs, a state space formulation for KMF control is required. The actuation equation in KMF control can be premultiplied by the pseudoinverse of *A*_1_, the actuation matrix:

$$w=A_{1}^{†}b_{1}.$$ (11)

This can then be substituted into the observation equation to yield the following:

$$b_{2}=A_{2}A_{1}^{†}b_{1}$$ (12)

Since Δ*x* (*k* + 1) = *b*_2_ and *u*(*k*) = *b*_1_, we can easily notice that:

$$\Delta x\left(k+1\right)=Bu\left(k\right)$$ (13)

where

$$B=A_{2}A_{1}^{†}$$ (14)

The cost function is defined as follows:

$$l\left(x,u\right)=∥x_{des}-x_{u}∥+∥u∥$$ (15)

where *x*_*des*_ is the desired state of the system. Formally, the optimization problem is expressed as follows:

$$\underset{u}{\min}\;J_{N}\left(x_{0},u\right)=\overset{N-1}{\underset{k=0}{\sum }}l\left(x\left(k\right),u\left(k\right)\right)$$ (16)

$$s.t.\;\Delta x\left(k+1\right)=Bu\left(k\right)$$ (17)

$$x\left(0\right)=x_{0}$$ (18)

$$∥u∥\leq u_{max}$$ (19)

$${\left(x_{1}\left(k\right)-a\right)}^{2}+{\left(x_{2}\left(k\right)-b\right)}^{2}\geq r^{2}$$ (20)

where *x*_0_ is the initial state of the system, *u*_max_ is the maximum torque to be applied, *x*_*i*_(*k*) is the *ith* system state variable, and {*a*, *b*, *r*} are the circular constraint parameters of the obstacle. Since the local linear model built is volatile and is only used for the next actuation, a linear MPC sufficies. As with all KMF controllers, stability and convergence are not guaranteed due to their exploratory nature.

## 4 Results

In this section, simulation and physical experimentation results are presented and discussed. In the first experiment, a simple reaching task on a simulated 2-DOF robot and a physical 2-DOF robot was performed using classical KMF controller and the proposed KMF-PC. In the second experiment, reaching tasks on a 2-DOF, 3-DOF, and 4-DOF simulated robot as well as the 2-DOF physical robot was performed with one and two circular obstacles. In the third experiment, the adaptability of the KMF-PC is demonstrated by modifying the 2-DOF robot’s kinematic structure midway. All the robots are planar, restricting the motion to the horizontal plane, to neglect the effects of gravity.

The simulation robots are simulated on MATLAB using Peter Corke’s Robotics Toolbox (Corke, 2017). The KMF-PC makes use of CasADi (Andersson et al., 2019). End-effector position was easily obtained using toolbox functions. On the other hand, the physical robot is comprised of aluminium links and Hebi X-Series actuators, (X5-9) capable of delivering 9 *Nm* of continuous torque, which are controlled using torque signals. The end effector of the robot is tracked using a calibrated webcam and a green marker. Direct pixel coordinates is used for determining the end-effector position for physical experiments. Virtual obstacles are used to emulate a circular obstacle that is to be avoided.

### 4.1 Experiment 1: Reaching Using Classical KMF and KMF-PC

**FIGURE 2:**
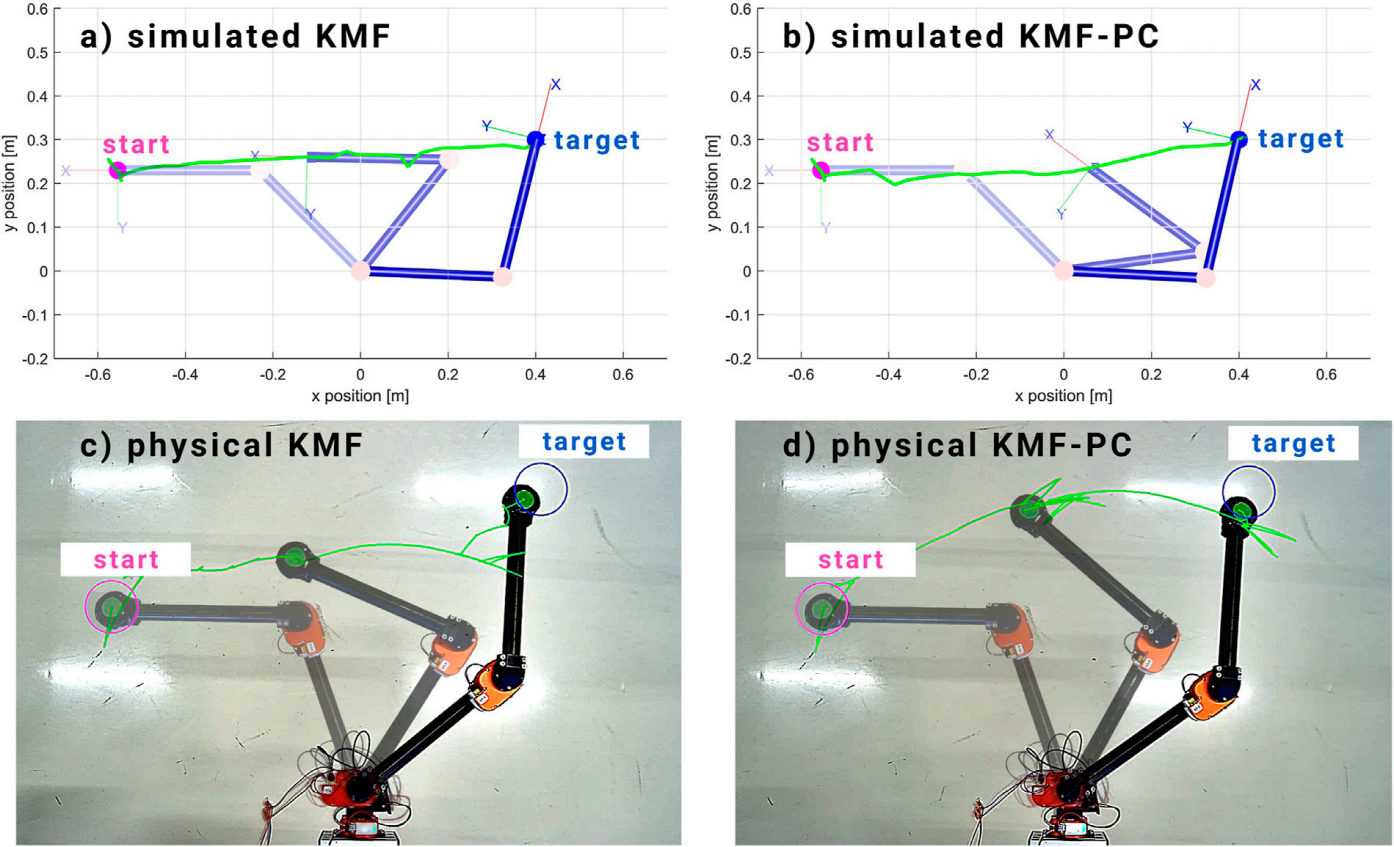
Simulated and physical experimentation of KMF control and the proposed KMF-PC in the absence of any obstacle. Comparing simulation results **(A,B)**, both controllers performed similar, reaching the target in a semi-straight line. As for the physical experiments **(C,D)**, we notice that the KMF-PC took a slightly more curved path to reach the target position. Nonetheless, both KMF and KMF-PC performed similarly well, achieving the reaching task within 30 steps.

In this experiment, classical KMF and the proposed KMF-PC are used to perform a simple reaching task on a simulated and a physical 2-DOF robot in the absence of any obstacle. Figure 2 shows both the simulated and physical experimentation results of running both controllers.

It the evident that, in the absence of any obstacle, both controllers performed similarly and were able to reach the target position in less than 30 steps.

### 4.2 Experiment 2: Obstacle Avoidance Using KMF-PC

**FIGURE 3:**
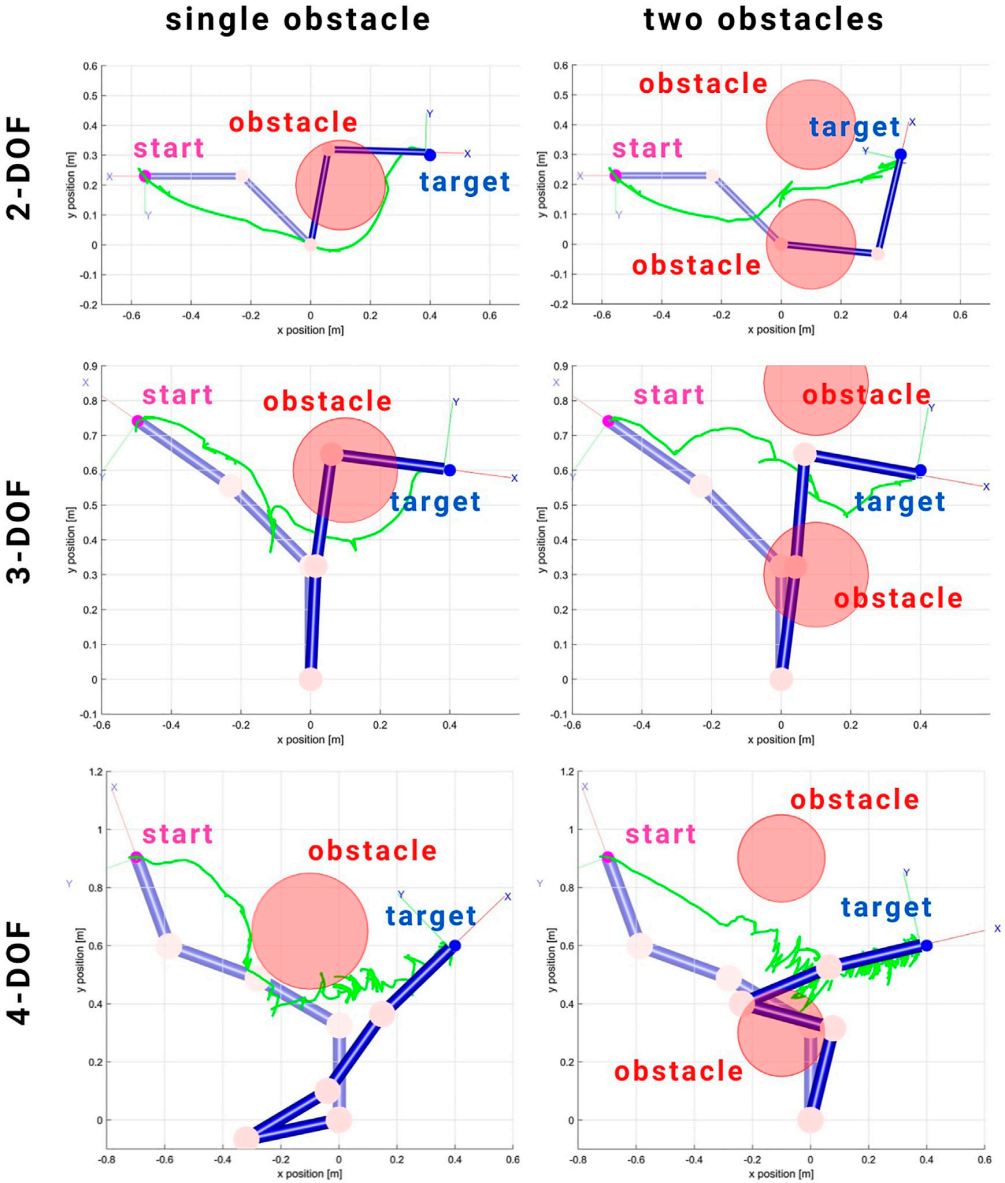
Simulation results of simultaneous reaching and obstacle avoidance using KMF-PC on simulated 2-DOF, 3-DOF, and 4-DOF manipulator robots. It is noted in the 4-DOF case, where the complexity is relatively higher, more exploratory motion is triggered which caused the end effector to penetrate the virtual obstacle.

In the second experiment, obstacle avoidance is performed on a 2-DOF, 3-DOF, and 4-DOF simulated robot and the 2-DOF physical robot. The results of simulations are summarized in Figure 3. The KMF-PC is capable of avoiding obstacles along the reaching path. Despite the increase in complexity when an extra DOF is added, the controller successfully performs the reaching task whilst avoiding the obstacles (2-DOF and 3-DOF). It also noted, due to the exploratory nature of the KMF approach, should the controller trigger an exploratory phases near any obstacle, this might cause collision with the obstacle (evident in the 4-DOF case). As such, it is recommended to inflate the obstacles to avoid any collision due to exploratory behaviour.

**FIGURE 4:**
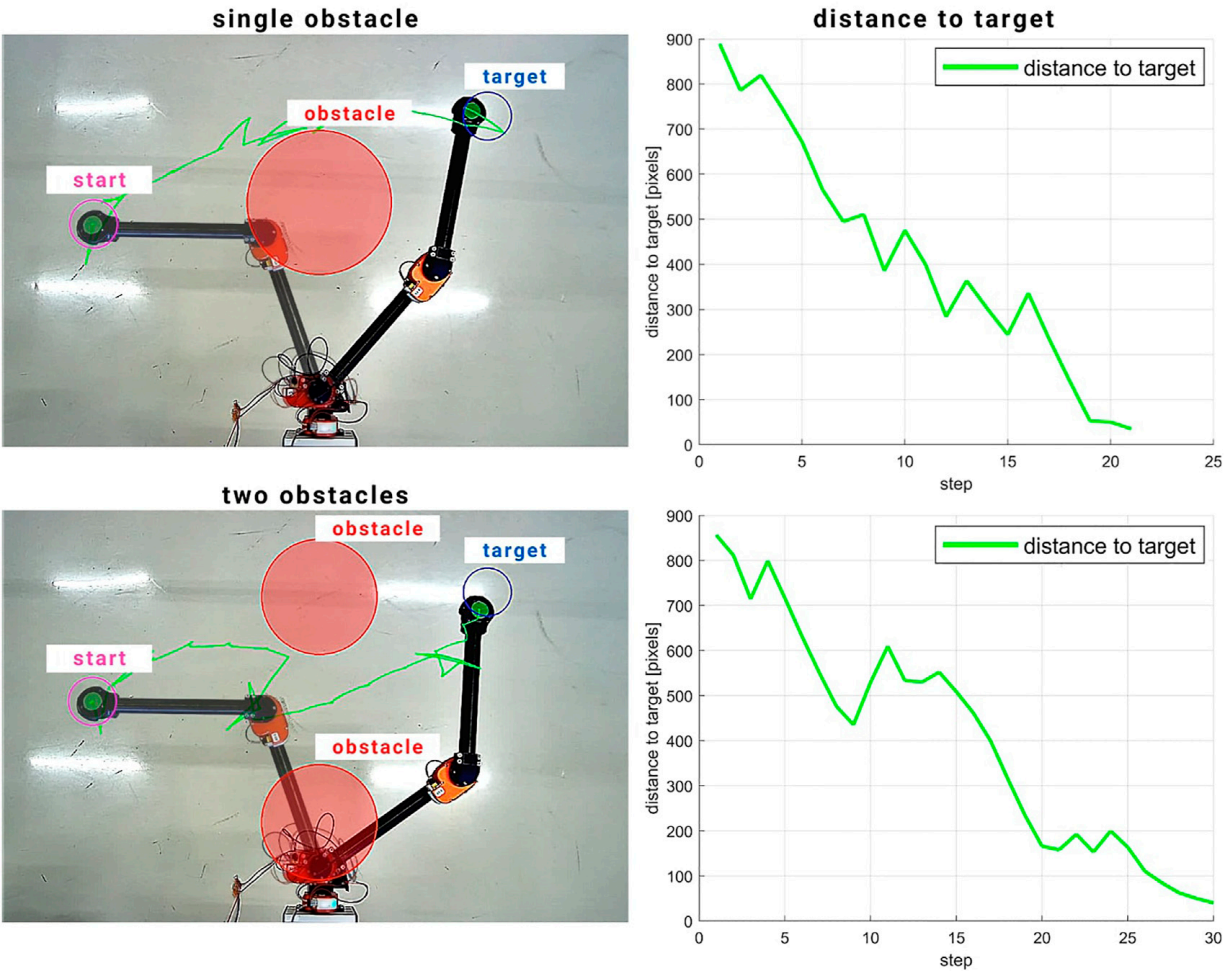
Physical experiment results of KMF-PC reaching and obstacle avoidance. In both cases, the end effector reached close to the obstacle. As such, obstacle inflation is recommended. In the single obstacle scenario, the robot reached the target in 21 steps. In the two obstacles scenario, it reached the target in 30 steps.

As for the physical experiments, the results are shown in Figure 4. The KMF-PC controller successfully performed the reaching task whilst avoiding the obstacles. In both cases, single and two obstacles, the KMF-PC controller brought the instances where the robot came close to the obstacle(s). As such, inflating the obstacles is recommended.

When one obstacle was presented, the controller took 21 steps to reach the target end-effector position. When two obstacles were in the way, the controller took 30 steps to reach the target.

### 4.3 Experiment 3: Adaptability of KMF-PC

**FIGURE 5:**
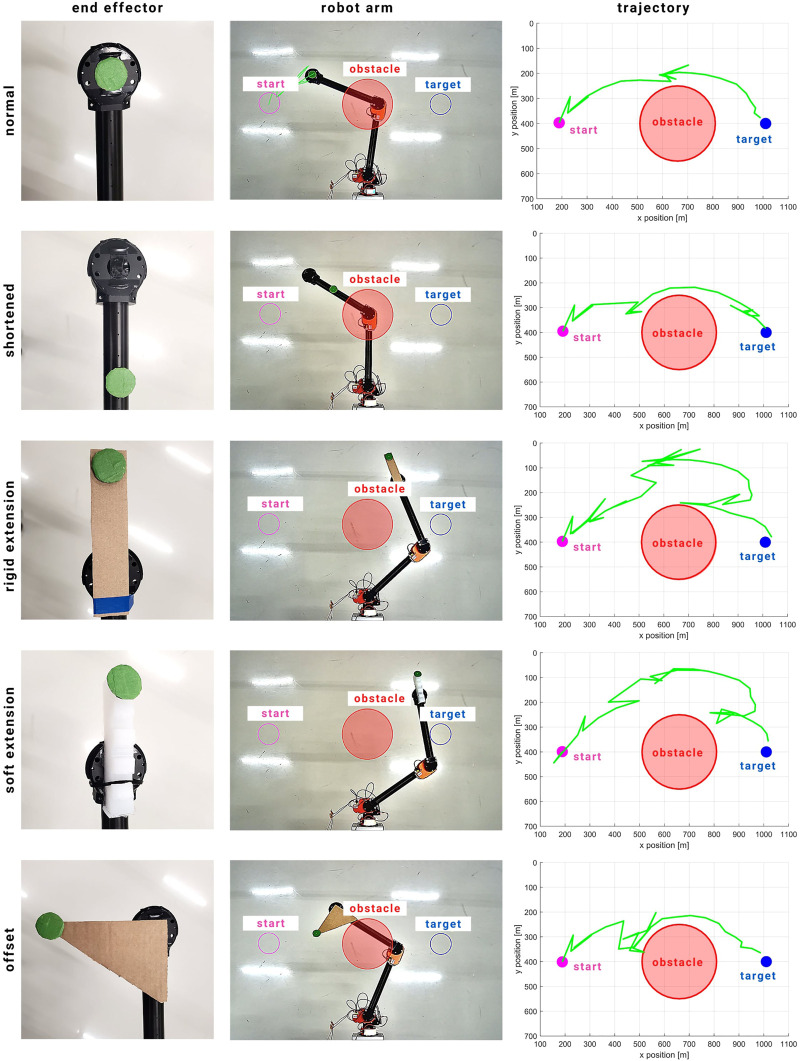
Physical experiment results of reaching and obstacle avoidance whilst modifying the end effector during execution. When the end effector is altered, the controller triggers an exploration phase and resumes reaching and obstacle avoidance right after. The modifications include shortening the end effector, rigid and soft extensions, and offsetting the end effector.

In the third experiment, the adaptability of the proposed controller is demonstrated through a series of tests in which the kinematic structure of the physical robot is modified midway the reaching task. The results are summarized in Figure 5. First, the robot’s second link is shortened by moving the marker down the link. Second, the link is extended by adding a rigid cardboard to the last link. Third, the end effector is extended using a soft link. Fourth, the end effector is offset.

The end effector is altered by pausing the execution temporarily and manually modifying the end effector. After the modification, the execution is resumed. In all cases, when the execution is resumed, the controller goes into an exploratory phase which is triggered due to the discrepancy measured between the predicted and actual state of the end effector. The controller, then after, resumes moving toward the target position. In all cases, the robot managed to reach the target within 50 steps.

## 5 Conclusion

A novel KMF controller is proposed that is capable of obstacle avoidance, using an MPC approach, without any prior knowledge of the robot’s model. KMF controllers make no assumption about the robot’s structure nor the environment. The formulation of the proposed KMF-PC was derived in this paper, showing how obstacle avoidance can be achieved without prior knowledge of the robot’s dynamics or kinematics. Using this approach to allow MPC to be applied to unknown robots enables a greater level of control to be achieved, with minimal computational complexity. Both simulated and physical experiments were conducted. Running the KMF-PC controller in the absence of any obstacle behaved similar to the classical KMF controller. Single and double obstacle avoidance was tested in simulation using 2-DOF, 3-DOF, and 4-DOF robots and on a physical 2-DOF robot. It is found that due to the exploratory nature of KMF control, any exploratory actuations trigger near any obstacle might cause the robot’s end effector to penetrate or collide with the obstacle. As such, obstacle inflation is recommended. Furthermore, the adaptability of the KMF-PC was demonstrated through a series of tests where the end effector was modified halfway during execution. In all experiments, the robot managed to perform simultaneous reaching and obstacle avoidance, reaching the target position in less than 50 steps. At the current state, KMF should be applied to robot control problems where flexibility and adaptability is favoured over accuracy and speed. Future work includes obstacle-aware exploratory actuations that take into account the obstacles to avoid collisions and continuous control to allow for gravity compensation and, thus, non-planar control.

## Data Availability

The datasets presented in this article are not readily available because the data generated was from simulation runs and physical experimentations on the robot. The data collected are summarized in figures in the article. Requests to access the datasets should be directed to a.alattar19@imperial.ac.uk.
